# Managing Robotic Radical Prostatectomy in Men with Penile Prosthesis: Surgical Technique, Outcomes, and Literature Review

**DOI:** 10.1590/S1677-5538.IBJU.2025.0467

**Published:** 2025-09-10

**Authors:** Rohan Sharma, Yu Ozawa, Shady Saikali, Avaneesh Kunta, Marcio Covas Moschovas, Travis Rogers, Vipul R. Patel

**Affiliations:** 1 Global Robotics Institute Advent Health Florida United States Global Robotics Institute, Advent Health, Celebration, Florida, United States; 2 University of Central Florida College of Medicine Florida United States University of Central Florida, College of Medicine, Florida, United States

**Keywords:** Prostatic Neoplasms, Penile Prosthesis, Robotic Surgical Procedures

## Abstract

**Introduction:**

Robotic-assisted radical prostatectomy (RARP) in patients with pre-existing inflatable penile prostheses (IPP) poses technical challenges due to the intrapelvic reservoir. With rising rates of prostate cancer and IPP use, evidence on safely performing RARP in this group is limited. This study assesses the feasibility, safety, and perioperative outcomes of RARP in men with prior IPP.

**Materials and Methods:**

We retrospectively analyzed 32 prostate cancer patients with functional three-piece IPPs who underwent RARP (2016-2024), excluding those with prior pelvic radiation, malleable implants, or incomplete data. Key adaptations included tailored port placement, cold dissection near the reservoir, site-specific retraction without reservoir removal, and intraoperative deflation as needed. Perioperative, functional, and oncologic outcomes were systematically assessed.

**Results:**

Median age was 67 years (IQR 61–73). Follow up duration was 24 months from RARP. Median operative time and blood loss were 110 minutes (IQR 98–120) and 100 mL (IQR 50–120), respectively. No intraoperative prosthesis injuries occurred. Clavien–Dindo grade I–II complications were observed in 8 patients (25%). Median time to continence (≤1 pad/day) was 56 days (IQR 46–92). All IPPs remained functional postoperatively without revision. 31 patients were continent at 12 months. Pathologic pT2 disease was present in 16 (50%) patients; positive margins occurred in 4 (12.5%) patients. Biochemical recurrence was noted in 9.4% at 12-month median follow-up.

**Conclusion:**

RARP in patients with a pre-existing penile prosthesis reservoir is technically feasible and safe, with no increase in procedure-related or reservoir-specific complications.

## INTRODUCTION

Inflatable penile prothesis (IPP) is the gold standard treatment with high satisfaction rates for severe or medically refractory erectile dysfunction (ED) across the World. United States is the leading country performing IPP followed by Germany and United Kingdom (1). According to the American Board of Urology statistics from 2003 to 2012, the utilization of inflatable penile prostheses (IPP) increased twelve-fold compared to malleable prostheses, with a reported IPP-to-malleable prosthesis ratio of 25:1 (2). At French database registry, there is 11-fold increase in use of IPP to malleable prosthesis from 2000-2013 (3). Median age group of patients with ED undergoing IPP implant is 64 years, according to the data reported by Weinberg et al. (4). Also, median age of patients undergoing robotic assisted radical prostatectomy (RARP) for prostate cancer (PCa) is 62 years (5). Given the rising incidence of ED and IPP placement, it is increasingly common to encounter patients with pre-existing IPPs requiring RARP.

A three-piece IPP consists of a reservoir, cylinders, and a pump. The reservoir is most commonly positioned in the Retzius space, which may render it susceptible to damage during dissection or space creation to assess the prostate, resulting in prosthesis malfunction or perforation (6, 7).

The existing literature provides limited evidence on surgical techniques or modifications for performing RARP in patients with a pre-existing penile prosthesis. This study outlines the surgical technique, along with key modifications and intraoperative strategies, to protect the penile prosthesis reservoir positioned in the Retzius space while ensuring the safe execution of RARP.

## MATERIALS AND METHODS

This study was conducted through retrospective data analysis retrieved from the AdventHealth Research Institute IRB (IRB 237998) approved Urologic Robotic Surgery Outcomes Registry.

### Study Endpoints

The primary endpoint of the study was to assess the perioperative safety of RARP in patients with a pre-existing penile prosthesis reservoir placed in the pelvis. Safety was evaluated based on intraoperative and postoperative complication rates measured by Clavien-Dindo grading, any prosthesis-related complications, and the need for prosthesis revision within 24 months postoperatively (8).

Secondary endpoints included functional outcomes, such as penile prosthesis performance at 3 months and urinary continence rates. Oncological outcomes were also evaluated and included final pathological analysis. Additional perioperative parameters, such as operative time, estimated blood loss, length of hospital stay, and readmission within 30 days, were also described.

### Study design and patient selection

This single-arm retrospective cohort study was conducted using data from the institutional prostate cancer registry between 2016 and 2022. Patients were followed for 24 months. A total of 32 patients diagnosed with PCa who underwent RARP with a pre-existing, functional three-piece penile prosthesis were included. Patients with a history of prior pelvic radiation therapy or a malleable penile prosthesis without a reservoir positioned in the Retzius space, salvage prostatectomy or incomplete follow up were excluded from the analysis.

Descriptive statistics were used to summarize baseline demographics, perioperative, and pathological variables. Continuous data were reported as medians with interquartile ranges (IQR) or means with standard deviations (SD), as appropriate. Categorical variables were presented as frequencies and percentages. Given the limited sample size, no inferential or comparative analyses were performed. Statistical analyses were conducted using R version 4.3.2 within the RStudio environment (RStudio, PBC, Boston, MA, USA).

### Surgical technique

**Figure 1:**
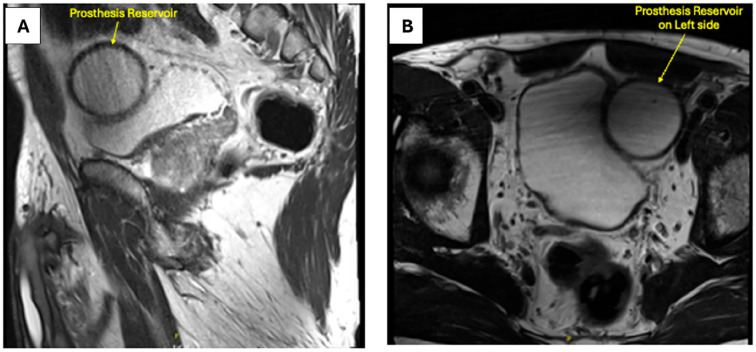
Representative preoperative pelvic magnetic resonance imaging (MRI) demonstrating the presence and location of a pre-existing penile prosthesis reservoir in patients undergoing robot-assisted radical prostatectomy.

All procedures were performed by a single experienced robotic surgeon (VP) using the da Vinci Surgical System^®^ via a standard transperitoneal six-port configuration, with minor adjustments to port placement based on the side and position of the inflatable penile prosthesis (IPP) reservoir (9-11). Reservoir location was assessed on preoperative MRI, as shown in Figure-1. Ports were typically shifted 2–3 cm medially and cranially on the side of the reservoir to reduce instrument collision and maintain optimal ergonomics. An anterior approach to RARP was uniformly employed. After establishing pneumoperitoneum, intra-abdominal inspection was performed to localize the IPP components, with particular attention to the reservoir, which was frequently found in the lateral pelvic wall or retropubic space.

A) Sagittal T2-weighted image showing a prosthesis reservoir in the retropubic space anterior to the bladder; B) Axial T2-weighted image showing a prosthesis reservoir positioned on the left side of the pelvis.

**Figure 2:**
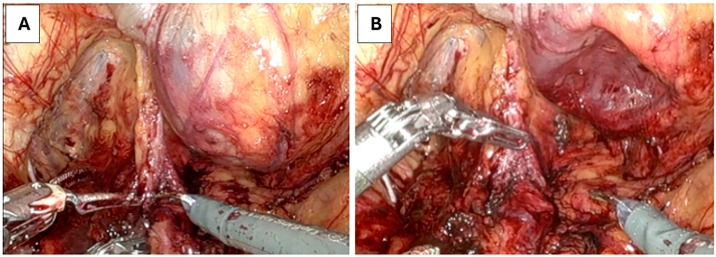
Intraoperative robotic images demonstrating the effect of cycling the penile prosthesis reservoir to facilitate pelvic dissection during robot-assisted radical prostatectomy.

Before starting the procedure, the IPP was tested to guarantee that it was appropriately working. During dissection, a cold-cut technique was employed to carefully mobilize the reservoir while preserving a surrounding pseudocapsule. This approach is intended to prevent direct instrument contact with the reservoir surface, thereby reducing the likelihood of microinjury and preserving prosthesis integrity. If the reservoir was seen overlying pelvic lymph node basins or obstructing the field, lymphadenectomy on that side was omitted to avoid iatrogenic prosthesis injury. Once bladder drop was complete, or earlier if the reservoir impeded dissection, the device was deflated by squeezing the scrotal pump to transfer fluid into the penile cylinders as shown in Figure-2. Standard RARP dissection proceeded with the IPP reservoir left undisturbed. After completion of the vesicourethral anastomosis, the reservoir was refilled to assess for any fluid leak or damage, ensuring device integrity before case completion.

A) Prior to cycling: the inflated reservoir occupies significant space in the pelvis, limiting surgical access and visualization; B) After cycling decompression of the reservoir creates additional working space, improving exposure of the bladder and anterior rectum.

**Figure 3:**
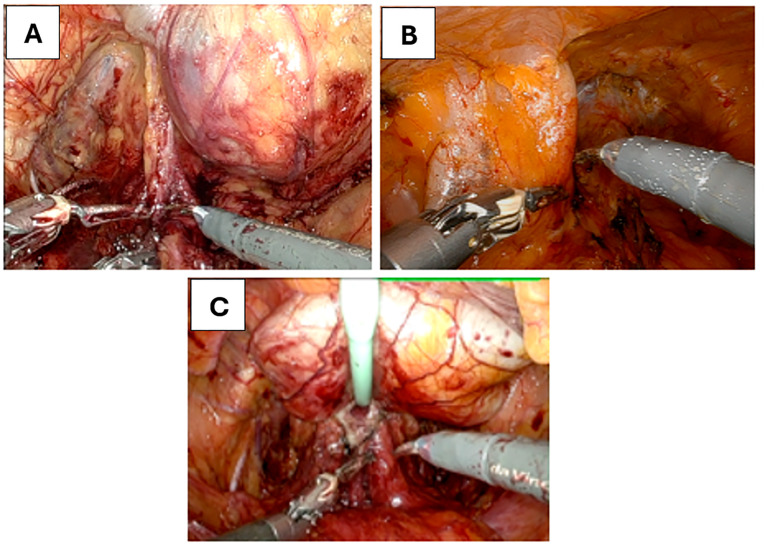
Intraoperative robotic views demonstrate various anatomical locations of pre-existing penile prosthesis reservoirs during robot-assisted radical prostatectomy.

**Figure 4:**
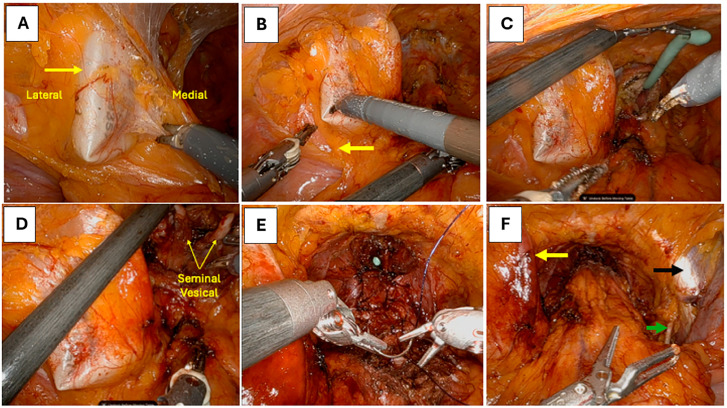
Intraoperative steps of robot-assisted radical prostatectomy in a patient with a pre-existing penile prosthesis reservoir located in the left pelvic (Retzius) space.

In cases where the reservoir was positioned on the right, the bedside assistant employed a suction instrument to retract the bulging reservoir as needed (Figure-3A). If the reservoir was located on the left side, the robotic fourth arm prograsp was utilized to retract the reservoir medially or superiorly without grasping, thereby improving exposure and facilitating dissection (Figure-3B). For reservoirs positioned in the midline, initial retraction was achieved by gently pulling up on the Foley catheter using the robotic fourth arm, which allowed the reservoir to be displaced posteriorly and lie between the pubic symphysis and the catheter itself (Figure-3C). This maneuver created a working space anterior to the reservoir. Following this, the fourth arm grasper or forceps was used directly to hold and retract the reservoir away from the operative field as shown in Figure-4. These site-specific retraction strategies enabled safe continuation of the bladder drop and prostate dissection while minimizing manipulation of the prosthesis and preserving its integrity.

A) Right-sided reservoir occupying the right pelvic space, visualized and retracted using the robotic fourth arm to optimize surgical exposure; B) Left-sided reservoir displaced laterally to facilitate bladder mobilization and dissection; C) Midline reservoir located posterior to the pubic symphysis; initial retraction achieved by elevating the Foley catheter to create a working plane posteriorly.

A) Medial and lateral dissection planes demonstrated on the left-sided prosthesis; careful dissection is carried out along the medial aspect, maintaining the pseudocapsule. The yellow arrow indicates the reservoir; B) Yellow arrow highlighting a pelvic lymph node located deep to the reservoir, obstructing access to the nodal basin; C) Elevation of the Foley catheter to facilitate bladder neck dissection; this maneuver creates a safe plane anterior to the reservoir and avoids direct compression; D) Lifting of the seminal vesicle to expose the posterior plane for safe dissection and nerve-sparing without difficulty.

(E) Vesicourethral anastomosis in progress; adequate space is maintained to prevent inadvertent needle injury to the underlying reservoir.

(F) Final view showing the reservoir (yellow arrow), the dissected right pelvic lymph node bed (black arrow), and the obturator nerve (green arrow) preserved laterally.

### Postoperative management

Patients were discharged on postoperative day one upon demonstrating comfort, tolerance of oral intake and Foley catheter remaining in situ. In most cases of RARP performed at our centre without an IPP, a single preoperative antibiotic dose was deemed sufficient, with an additional single dose of levofloxacin administered one day prior to catheter removal. However, in patients with a pre-existing inflatable penile prosthesis (IPP), a daily dose of levofloxacin was continued until catheter removal. Patients were advised to resume inflating and deflating the IPP after 1 month, once daily. Patient could resume the regular use of IPP after 3 months.

### Literature review

We performed a focused literature search using PubMed, Embase, Scopus, and Google Scholar to identify relevant studies published up to March 2025. Search terms included combinations of "robotic prostatectomy," "inflatable penile prosthesis," "reservoir," and "perioperative outcomes." This review was undertaken to contextualize our study, refine our operative approach, and select outcome measures in line with existing evidence.

## RESULTS

A total of 32 patients were included. Baseline demographic and clinical characteristics are summarized in Table-1. The median age was 67 years (IQR 61–73), and the median body mass index (BMI) was 27.8 kg/m^2^ (IQR 26.2–30.8). Comorbidities included diabetes mellitus in 25% of all the cohort, hypertension in 34%, coronary artery disease in 9%, and hyperlipidemia in 47%. Ten patients (31.2%) reported a history of smoking. The median preoperative prostate-specific antigen (PSA) level was 6.3 ng/mL (IQR 3.9–9.4). A palpable nodule on digital rectal examination was present in 68% of patients. On prostate biopsy, Gleason Grade Group (GG) 2 and 3 tumors were most common (53.1% and 18.8%, respectively). Mean prostate volume was 64 ± 26.9 cc. Based on D’Amico risk stratification, most patients had intermediate-risk disease (68.8%).

**Table 1:** Baseline demographic and clinicopathological characteristics of patients undergoing robot-assisted radical prostatectomy with pre-existing penile prosthesis.

Variable		Value
Number of patients		32
Age, years Median (IQR)		67(61-73)
Body Mass Index (BMI), kg/m^2^ Median (IQR)		27.8(26.2-30.8)
**Comorbidity (Present/Absent)**		
Diabetes Mellitus		8/24
Hypertension		11/21
Coronary Artery Disease		3/29
Hyperlipidemia		15/17
Smoking History (Present/Absent)		10/22
Preoperative PSA, ng/mL Median (IQR)		6.3(3.9-9.4)
**DRE Palpable nodule, n (%)**		
	Yes	20 (68.8%)
	No	12 (31.2%)
**Biopsy ISUP Gleason Grade Group, n (%)**		
	GG1	3 (9.4%)
	GG2	17 (53.1%)
	GG3	6 (18.8%)
	GG4	4 (12.5%)
	GG5	2 (6.3%)
Prostate volume cc (Mean ± SD)		64 ± 26.9
**D´Amico Classification, n (%)**		
	Low	4 (18.9%)
	Intermediate	22 (68.8%)
	High	6 (12.5%)
**Final Pathology ISUP GG, n (%)**		
	GG1	1(3.13%)
	GG2	16(50%)
	GG3	9(28.13%)
	GG4	1(3.13%)
GG5		
**Pathological T staging, n (%)**		
	pT2	16(50%)
	pT3a	13(40.6%)
	pT3b	3(9.4%)
Extraprostatic Extension (Yes/No)		16/16
Seminal Vesical Invasion (Yes/No)		3/29
**Pathological N staging**		
	N0	16
	N1	1
	Nx	15
Perineural Invasion (Yes/No)		26/6
Lymphovascular Invasion (Yes/No)		5/27
Positive Surgical Margins (Yes/No)		4/28
Tumor Volume percentage, cc Median (IQR)		10(5-20)
Tumour diameter, cm Median (IQR)		1.5(1.1-1.9)
Number of Patients with BCR		3
Number of days to BCR, in days Mean		373

Perioperative outcomes and postoperative complications are presented in Table-2. The median total operative time was 110 minutes (IQR 98–120), with a median console time of 90 minutes (IQR 78–90). Full bilateral nerve-sparing was achieved in 34.4% of patients, while partial nerve-sparing was performed in 56.3%, and no nerve-sparing in 6.3%. There were no intraoperative injuries to the penile prosthesis in any patient. The median estimated blood loss was 100 mL (IQR 50–120). No patients experienced intra- or postoperative sepsis, pelvic hematoma requiring transfusion, or required surgical re-exploration.

**Table 2:** Perioperative outcomes and complication rates following robot-assisted radical prostatectomy in patients with pre-existing penile prosthesis.

Variable		Value
Total OR time, minutes Median (IQR)		110(98-120)
Total Console time, minutes Median (IQR)		90(78-90)
**Degree of Nerve Sparing, n (%)**		
	Non-Nerve Sparing	2 (6.25%)
	Partial Nerve Sparing	18 (56.25%)
	Full Nerve Sparing	11 (34.38%)
**Clavien-Dindo Grading**		
	1	6
	2	2
	3	0
	4	0
	5	0
Intra operative Prosthesis injury		None
Estimated Blood Loss, cc Median (IQR)		100(50-120)
Post op Sepsis		None
Number of cases with Post operative Lymphocele		6 (18.75%)
Post op Lymphocele requiring Intervention		None
Post op pelvic Hematoma requiring Transfusion		None
Post op re exploration		None
Total Hospital stay, days Median (IQR)		1(1-1)
Post operative catheter, days Median (IQR)		6(5-7)
Number of days to Continence, Median (IQR)		56(46-92)

Asymptomatic postoperative lymphocele occurred in 6 patients (18.8%), none of whom required intervention. According to the Clavien-Dindo classification of surgical complications, 6 patients experienced Grade I complications, and 2 patients experienced Grade II complications; there were no Grade III, IV, or V complications. The median length of hospital stay was 1 day (IQR 1–1), and the median duration of postoperative catheterization was 6 days (IQR 5–7).

Final pathological findings are detailed in Table-1. Pathologic GG 1–3 tumors were identified in 81.3% of patients, with GG 4 or 5 tumors in 15.6%. Organ-confined disease (pT2) was present in 50%, while 40.6% of patients had extracapsular extension (pT3a), and 9.4% had seminal vesicle invasion (pT3b). Perineural invasion was observed in 81.3% of specimens. Positive surgical margins were identified in 12.5% of cases. The median tumor volume was 10% (IQR 5–20), and the median tumor diameter was 1.5 cm (IQR 1.1–1.9). At the 24 months follow up duration, 3 patients had developed biochemical recurrence (BCR).

The median time to recovery of continence (defined as pad-free or ≤1 pad/day) was 56 days (IQR 46–92). All patients maintained preserved function of the penile prosthesis at 3 months postoperatively, with no instances of mechanical malfunction, revision surgery, or explantation.

## DISCUSSION

In our study, we assessed the feasibility and safety of robotic-assisted RARP in patients with pre-existing three-piece inflatable penile prostheses (IPP). We demonstrated favourable outcomes, with effective preservation of IPP integrity and functionality, satisfactory continence rates, and acceptable oncological control. These results affirm that with meticulous surgical planning and careful intraoperative techniques, RARP can be successfully performed in this specialized patient population.

To contextualize our results, we conducted a literature review summarizing existing studies of RARP in men with pre-existing IPP. The largest study by Razdan et al. (2024) included 155 patients undergoing RARP with pre-existing IPP. This study notably reported no instances of IPP injury, achieving complete continence in 100% of patients by 10 months postoperatively, and a BCR rate of just 1.9%. Razdan et al. described two primary intraoperative approaches to reservoir management: the "No Touch Technique," preserving the reservoir's pseudocapsule intact, and the "Safe Mobilization Technique," involving relocation of the reservoir away from the operative field. Both approaches were associated with minimal complications, reflecting their viability and safety (12). In our cohort, we performed all the cases with "No touch technique", where we avoided dissection lateral to the reservoir while medial dissection was performed using cold cuts to prevent microinjury. Our study cohort had three patients having BCR and one patient having incontinence over 24 months follow up. Although all the prosthesis remained functional during entire duration of follow up without requiring any intervention.

Smaller studies provide additional insights into surgical technique variations and outcomes. Erdeljan et al. (2011) reported successful outcomes in two patients employing a pelvic reservoir-sparing technique. Both patients maintained IPP function without complications, had negative surgical margins, and showed no BCR at three months (13). Similarly, Kyung et al. (2010) described a single case managed using a deflation-inflation reservoir technique, achieving pad-free continence at six months and no BCR (14).

Sulman et al. (2008) detailed two patients who underwent a careful reservoir deflation approach with meticulous preservation of the reservoir's pseudocapsule (15). Both surgeries concluded without complications or prosthesis damage, underscoring the importance of precise technique and the careful handling of IPP components. Finally, Yenice et al. (2020) uniquely described a robotic perineal radical prostatectomy approach in a single patient, highlighting an alternative route that completely avoids interference with the prosthesis reservoir located in the retropubic space. The operation was completed successfully, without IPP-related complications or reservoir injuries (16).

Alternative surgical approaches, such as the robotic perineal approach described by Yenice et al. (2020), may be advantageous in select scenarios, particularly when reservoir manipulation poses excessive risks (16). The transperitoneal approach for RALP in patients with pre-existing IPPs is safe, effective, and associated with favourable continence and device outcomes. Unlike the perineal route, it offers broader clinical experience and fewer risks related to reservoir manipulation, supporting its role as the preferred surgical technique in this setting (12).

Our study and existing literature demonstrate that RARP can be safely performed in patients with pre-existing IPPs without compromising functional or oncologic outcomes. Intraoperative strategies should be individualized based on reservoir location, pelvic anatomy, body habitus, and surgical history. Preoperative counselling is essential to discuss risks such as prosthesis malfunction, infection, and rare reservoir revision. Surgeons should also inform patients that ipsilateral lymph node dissection may be limited due to the risk of reservoir injury.

Given the increasing prevalence of both PCa and ED in the aging male population, the clinical scenario of concurrent IPP and PCa management is likely to become more frequent. Hence, patient workup prior to IPP procedure, should aim to rule out PCa or any family history of PCa (17, 18). Considering patient having high risk of PCa or pelvic cancer in future requiring any form of surgical intervention, surgeon may consider placing the reservoir in ectopic position (19-21). Establishing standardized guidelines and adopting the described reservoir management strategies into surgical protocols can further optimize patient outcomes and surgeon confidence.

This retrospective, single-surgeon study is subject to selection bias, variable follow-up, and limited external validity. The small cohort and absence of a control group precluded comparative analysis. However, it represents one of the largest series on RARP in men with penile prostheses, enhancing interpretability relative to prior reports. Our comprehensive review of the literature provides important context for the observed outcomes. While the findings are hypothesis-generating due to the study's design and short follow-up, they highlight the need for prospective, multicenter studies to validate surgical techniques and outcomes in this unique patient population.

## CONCLUSIONS

RARP can be performed safely in patients with a pre-existing penile prosthesis reservoir located in the pelvis. Preoperative imaging, particularly MRI, plays a critical role in delineating the reservoir's position relative to the prostate and lymph node basins, enabling accurate risk stratification and patient counselling regarding the feasibility of lymph node dissection. Intraoperatively, meticulous dissection while preserving the pseudocapsule around the reservoir minimizes the risk of prosthesis injury. Tailored trocar placement can facilitate direct access to key anatomical structures, allowing the procedure to be performed with minimal compromise to the surgical approach. These findings reinforce the procedural feasibility of RARP in this select population and underscore the necessity of surgical experience and thoughtful technical adaptation to maintain operative safety.

## Data Availability

The data underlying this article are not publicly available due to patient privacy considerations but may be shared upon reasonable request from the corresponding author and with appropriate institutional approvals.

## ABBREVIATIONS

IPP: Inflatable Penile Prosthesis
ED: Erectile Dysfunction
RARP: Robotic assisted radical prostatectomy
Grade Group: GG
PCa: Prostate cancer
PSA: Prostate Specific Antigen
GG: Gleason Grade
DRE: Digital Rectal examination
BMI: Body Mass Index
EBL: Estimated blood loss
BCR: Biochemical recurrence
IQR: Inter quartile range
SD: Standard deviation
MRI: Multiparametric resonance imaging

## DATA AVAILABILITY

All data generated or analysed during this study are included in this published article
